# Bioactive Compounds in Waste By-Products from Olive Oil Production: Applications and Structural Characterization by Mass Spectrometry Techniques

**DOI:** 10.3390/foods10061236

**Published:** 2021-05-29

**Authors:** Ramona Abbattista, Giovanni Ventura, Cosima Damiana Calvano, Tommaso R. I. Cataldi, Ilario Losito

**Affiliations:** 1 Chemistry Department, University of Bari Aldo Moro, via Orabona 4, 70126 Bari, Italy; ramona.abbattista@uniba.it (R.A.); giovanni.ventura@uniba.it (G.V.); tommaso.cataldi@uniba.it (T.R.I.C.); 2 Interdepartmental Centre SMART, University of Bari Aldo Moro, via Orabona 4, 70126 Bari, Italy; 3 Pharmacy-Pharmaceutical Sciences, University of Bari Aldo Moro, via Orabona 4, 70126 Bari, Italy

**Keywords:** LC-ESI-MS, MALDI-MS, bioactive phenolics, olive oil, olive leaves, olive pomace, olive oil mill wastewater

## Abstract

In recent years, a remarkable increase in olive oil consumption has occurred worldwide, favoured by its organoleptic properties and the growing awareness of its health benefits. Currently, olive oil production represents an important economic income for Mediterranean countries, where roughly 98% of the world production is located. Both the cultivation of olive trees and the production of industrial and table olive oil generate huge amounts of solid wastes and dark liquid effluents, including olive leaves and pomace and olive oil mill wastewaters. Besides representing an economic problem for producers, these by-products also pose serious environmental concerns, thus their partial reuse, like that of all agronomical production residues, represents a goal to pursue. This aspect is particularly important since the cited by-products are rich in bioactive compounds, which, once extracted, may represent ingredients with remarkable added value for food, cosmetic and nutraceutical industries. Indeed, they contain considerable amounts of valuable organic acids, carbohydrates, proteins, fibers, and above all, phenolic compounds, that are variably distributed among the different wastes, depending on the employed production process of olive oils and table olives and agronomical practices. Yet, extraction and recovery of bioactive components from selected by-products constitute a critical issue for their rational valorization and detailed identification and quantification are mandatory. The most used analytical methods adopted to identify and quantify bioactive compounds in olive oil by-products are based on the coupling between gas- (GC) or liquid chromatography (LC) and mass spectrometry (MS), with MS being the most useful and successful detection tool for providing structural information. Without derivatization, LC-MS with electrospray (ESI) or atmospheric pressure chemical (APCI) ionization sources has become one of the most relevant and versatile instrumental platforms for identifying phenolic bioactive compounds. In this review, the major LC-MS accomplishments reported in the literature over the last two decades to investigate olive oil processing by-products, specifically olive leaves and pomace and olive oil mill wastewaters, are described, focusing on phenolics and related compounds.

## 1. Introduction

**Figure 1:**
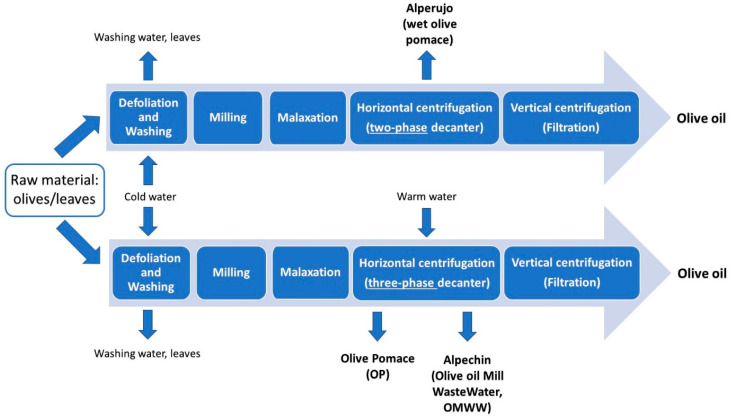
Layout of the main steps of olive oil production involving two- or three-phase horizontal centrifugation and the main by-products obtained.

The production of high-quality olive oils implies the generation of vast quantities of solid residues and/or wastewaters that may have a great impact on terrestrial and aquatic environments because of their high phytotoxicity [1]. Depending on the techniques used for olive oil production, namely, on the type of horizontal centrifugation (two-phase or three-phase), the process most adopted to separate olive oil from olive paste obtained after malaxation, different by-products are obtained (Figure 1).

When two-phase decanters are used for horizontal centrifugation, whereby water is not purposely added to the olive paste resulting from malaxation, a humid semi-solid pomace is obtained as by-product (wet olive pomace, wet OP, or *alperujo*), consisting in olive pulp and husk, crushed olive stone and aqueous solution. In the case of three-phase centrifugal technology, solid OP and olive oil mill wastewater (OMWW) or *alpechin* are the two major by-products. Notably, the modern two-phase centrifugal extraction technology merges OP with OMWW to produce a single by-product named olive mill waste (OMW) [2]. OMW shows a broad spectrum of toxicity, antimicrobial activity, high acidity and phytotoxicity, that make its biological degradation challenging and its disposal problematic for major producing countries (all located in the Mediterranean basin), given the significant amounts of OMW generated every year. Research efforts have thus been focused on valorizing the OMW in various ways, such as using it as energy source [3] or as fertilizer, biomass, or additive in animal feed [4]. However, a large quantity of by-products resulting from olive oil production remains unexploited, with their potentially high biological value being neglected. Indeed, wastes remaining after the production of olive oil are a heterogeneous mixture of many chemical components, such as metal ions, carbohydrates, and polyphenols, that may exert different biological activities, primarily acting as antioxidants [5]. Phenolic compounds from olive fruits are mostly retained in the corresponding waste, whereas just a small percentage (ca. 2%) is transferred to olive oils during production, thus making by-products an interesting alternative source of natural antioxidants for application in the food and pharmaceutical industries [5,6]. In the last few years, health benefits related to the assumption of novel olive by-products-based preparations have encouraged the research to identify the main compounds recognized for their positive physiological effects. In this context, the phenolic composition of olive oil production by-products has been extensively studied and several authors have applied chromatographic methods for their isolation and analysis, such as reversed-phase liquid chromatography (RPLC) coupled to mass spectrometry (MS) [7,8], fractionation by solid-phase extraction (SPE) and LC retention-time identification [9,10], preparative HPLC and capillary zone electrophoresis (CZE) with UV diode array detection (DAD) [11]. As emphasized by researches dealing with one of the most important classes of bioactive compounds related to olive oil, i.e., *secoiridoids* (see, for example, ref. [12]), MS provides the analytical strength for a highly detailed structural characterization, including the distinction of isomeric compounds.

After a survey on the general features and the main applications reported so far for olive oil by-products, including olive leaves, pomace, and olive mill wastewater, with a special emphasis on the correlation between their content of high-added value compounds and their reuse, this review will focus on the literature concerning the use of MS techniques for the extended characterization of the most relevant bioactive compounds in these matrices. Quantitative data, where available, obtained on specific compounds will be also reported and discussed in the perspective of individuating which compounds (or class of compounds) may deserve the development of appropriate recovery strategies from olive oil production by-products.

## 2. High-Added Value Compounds in Olive Oil Production Wastes

### 2.1. Olive Leaves

**Figure 2:**
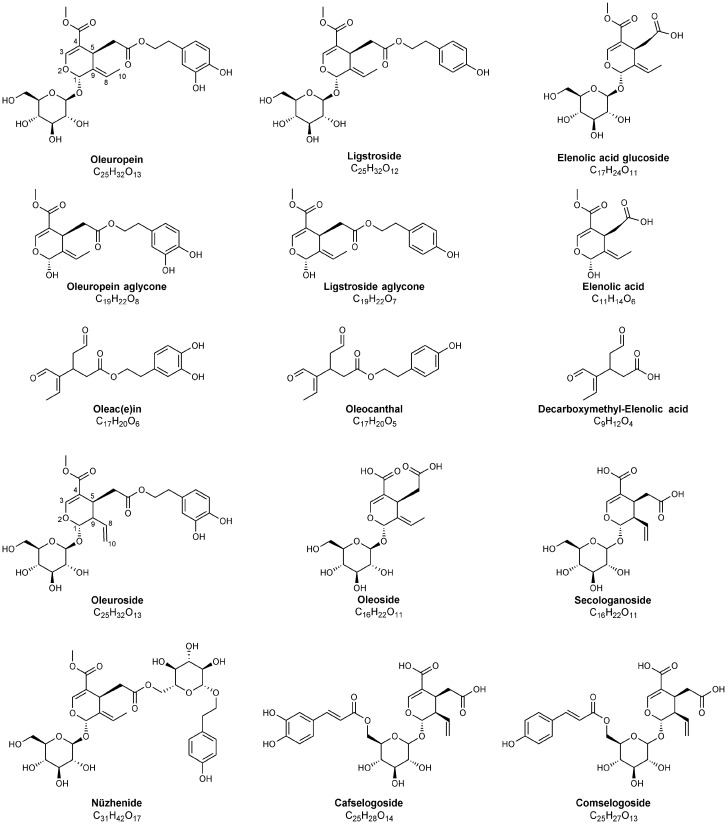
Chemical structures of major secoiridoids identified in olive leaves, pomace and in olive oil mill wastewater using MS techniques. Note that hemiacetalic forms of elenolic acid and of oleuropein and ligstroside aglycones are reported; dialdehydic open-structure isoforms are indicated for oleac(e)in, oleocanthal and decarboxymethyl-elenolic acid. The carbon atoms numbering typically adopted for secoiridoids has been reported for oleuropein and oleuroside to emphasize the positional isomerism occurring for these compounds, related to the position of the exocyclic C=C bond.

Olive leaves represent an agricultural waste by-product obtained during the harvesting of olive trees for table olives and olive oil production chains [13,14]. It has been estimated that pruning alone produces annually 25 kg of waste represented by branches and leaves per olive tree [15]. A considerable amount of olive leaves is also discarded during the olive drupes washing process at the beginning of the olive oil production chain (see Figure 1), reaching a value ranging from 8% [16] to 10% [17] of the total weight of olives subjected to milling. In general, olive leaves are not included in the definition of olive mill solid waste [18] but, together with olive stones, they are described as solid residue [19]. The use of solid residue is of great economic and social importance for the Mediterranean area, as it is accumulated in large amounts [19]. Although limited by their pungency, olive leaves are traditionally used in many countries as feed for livestock or simply disposed by burning. Olive leaves represent a potential source of bioactive compounds, as indirectly proved by the use of their extracts in the context of folk medicine for centuries, with the first medicinal application attested in history dating back to the ancient Egyptian civilization [13]. During centuries, the use of olive leaves preparations in traditional medicine has spread in many different countries [20] (see Table 1). Recently, preparations based on olive leaves, in the form of liquid extracts or tablets, have been commercialised as natural supplements against diabetes, high blood pressure, cardiovascular diseases, urinary tract infections, chronic fatigue symptoms and to improve immune system [21]. Olive leaves preparations also find their application in the cosmetic industry for their anti-ageing activity, and due to their antibiotic and antiparasitic properties as supplements for animal health [22]. Moreover, olive leaves extracts may be used as additives to increase food shelf-life, safety and functionality for their antioxidant and antimicrobial features [23,24]. Interestingly, their probiotic properties have been recently found to promote the *Lactobacillus casei* survival during cold storage of cheese [25]. Their addition to beer [26] and to a Southern Italy traditional cereal-based baked product known as *taralli* [17] has been found to increase the inherent polyphenols content of these products. It is also worth noting that phenolic compounds and tocopherols, normally found in olive oils, play a protective role against oxidative stress [27] and are able to extend the extra virgin olive oil shelf-life due to their antioxidant properties [28]. Since the use of synthetic antioxidants may lead to health risks, recent papers have featured that olive leaves, having a high antioxidant activity [29,30,31] due to their phenolics, can exhibit strong preventive effects against olive oil oxidation. The addition of olive leaves during oil extraction process, specifically during olive milling, has thus been recently evaluated in detail and found to lead to the enrichment in chlorophyll [32,33], carotenoids, flavonoids, and in the derivatives of oleuropein, which is the main secoiridoid occurring in olive leaves (see Figure 2) [34].

The promising antimicrobial and antioxidant hallmarks of phenols contained in olive leaves by-products have also led to produce bio-active films for food packaging by integrating olive leaf extract or powder in plastic polymers [44,45].

### 2.2. Olive Pomace and Olive Oil Mill Wastewater

According to Decision 2000/532/EC of the European Union Commission and to the Eurostat database classification [46], waste from agricultural activities (i.e., corn, wheat, fruit, vegetables, rice, pomace, olive wastes) are included in the agricultural livestock waste (ALW) category. Just to give an idea of the amount of ALW in 2016 resulting from olive oil production, 22 and 14 thousands of tons of olive pomace were generated, respectively, in Apulia and Sicily, two of the major olive oil producers in Italy [47]. Such amounts are not surprising, since approximately 800 kg of pomace and 200 kg of wastewater are on average obtained for 200 kg of extra virgin olive oils. To avoid endangering human health and to protect the environment, a rational waste management including prevention, reuse, recycling, recovery, and disposal is crucial also for such by-products [48].

As previously mentioned, the type of horizontal centrifugation adopted during olive oil production can determine the main features of OP, which is humid and semisolid, with a moisture content between 50 and 70%, when two-phase decanters are used, whereas moisture is decreased if three-phase decanters (35–40%) or traditional press mills (20–25%) are employed [49]. The average rough composition of OP has been reported as follows: water (60–70%), lignin (13–15%), cellulose and hemicellulose (18–20%), olive oil retained in the pulp (2.5–3%) and mineral solids (2.5%) [2]. Major organic compounds are sugars (3%), volatile fatty acids (C2–C7) (1%), poly-alcohols (0.2%), proteins (1.5%), polyphenols (0.2%) and pigments (0.5%) [2]. The profile vitamin E occurring in OP includes α-tocopherol, β-tocopherol, α-tocotrienol and γ-tocopherol, being α-tocopherol the major form (>2.6 mg/100 g). The lipid fraction is particularly rich in oleic acid (ca. 75%), followed by palmitic, linoleic, and stearic acids; hydroxytyrosol and comselogoside represent around the 80% of the total phenolics in OP [50].

Since OP consists essentially of olive pulp, olive stone and vegetation water, it includes many of the valuable components of the olive fruit so it can be subjected to biorefining, i.e., to the extraction of valuable compounds and energy. OP is mostly used for the recovery of pomace oil by solid-liquid extraction with hexane, followed by distillation and solvent recycling. The crude oil is then refined and typically blended with a small amount of virgin olive oil. Furthermore, after OP is evaporated and thermally concentrated, it can be applied to cultivated soils as herbicide, insecticide, and compost. Indeed, due to the polysaccharide occurrence, dried OP represents a potential source of gelling pectic material. As an example, Lama-Munoz et al. isolated sugars from *alperujo* by ethanol/water precipitation, obtaining various oligosaccharide fractions as pectin, neutral and acidic xylo-oligosaccharides, and xyloglucan oligosaccharides with diverse applications as gelling agents, stabilizers and emulsifiers for the food industry [51]. *Alperujo* compost at different doses was evaluated by Tortosa et al. [52] as an organic manure, mixed with a minimal amount of inorganic fertilizers, for pepper growth in greenhouse cultivations. The authors speculated that the organic matter from *alperujo* compost positively affects pepper oxidative metabolism by increasing the antioxidative enzymatic activities. Hence, the yield and physiological growth of plants would be improved or rather comparable to standard nutrient solutions. OP is also considered a source of bioethanol, biogas, and methane [53]; residuals of olive stones in OP can be the substrate to obtain activated carbon that can be employed as fuel for the generation of heat and electricity [19]. Moreover, *alperujo* oil has been used as a non-edible substrate with a high-free fatty acid content for the synthesis of biodiesel through an enzymatic path based on recombinant 1,3-positional specific *Rhizopus oryzae* lipase, avoiding the generation of glycerol as a co-product [54]. Another fascinating alternative of OP valorization is its use as a substrate for growing microorganisms of biotechnological interest [55]. This strategy offers the opportunity of producing high value-added products, such as enzymes, biopolymers, and pigments with a concomitant reduction of organic wastes. Ghilardi et al. explored the possibility to produce carotenoids by a strain of *Rhodotorula mucilaginosa* using different media derived from *alperujo* as a cheap substrate [55]. According to the medium used, it was possible to obtain a mixture of carotenoids enriched in torulene, torularhodin and/or neurosporene, thus showing that *alperujo* can be used to produce carotenoids exploitable at industrial scale, as additives in pharmaceutical, food and feed products [55].

OP has been applied even in the construction field. De la Casa et al. [56] reported the addition of *alperujo* to the ceramic paste of bricks, lowering density and thermal conductivity. The OP residue was also used as a substitute for clay in 1.25, 2.5 and 5% (wt) of artificial lightweight aggregate manufactures [57]. The results indicated that the addition of OP in the mixture is beneficial in terms of environmental impact compared to that related to aggregates made with clay.

Besides current uses, including composting, soil amendment, animal feed, OP can be considered a valuable source of bioactive substances with well-recognized benefits for human health and well-being. As aforementioned, it is rich in phenolic compounds and triterpenic acids, for which numerous biological activities, including anti-inflammatory, antitumor, antimicrobial, antioxidant, antidiabetic, and cardio-protective activities, have been reported [58]. Among bioactive phenols, hydroxytyrosol (HT) stands out among compounds with highest added value that can be recovered from the solid by-product, due to its high oxidative stability and antioxidant activity and it is currently used as a therapeutic agent, dietary supplement or natural ingredient in food and feed industries. For instance, various contents (65–195 μg) of HT were added to 100 g of fresh prepared mayonnaise and several quality parameters as free acidity, peroxide value and concentration of conjugated dienes, as well as the content of polyphenols and squalene in the lipid fraction [59], were evaluated [60]. The authors demonstrated that HT improves the mayonnaise hydrolytic stability reducing the formation of oxidation by-products during storage at room temperature and in the dark up to four weeks. Antioxidant-rich extracts from olive mill pomace related to tree different cultivars of Southern Italy (*Nocellara*, *Roggianella* and *Carolea*) were dissolved in a commercial pear juice using inulin as a carrier system of bioactive compounds [61]. In vitro evaluation highlighted outstanding antioxidant features of fortified juice in terms of both antioxidant and scavenging performances, representing an attractive source of functional foods. Very recently, Di Nunzio et al. [62] prepared bakery products (biscuits and breads) using a variety of flours and fermentation protocols also enriched with defatted OP. To assess the effects in a biological system, the digested fractions were supplemented to intestinal cultured enterocytes cells, used as model system, and the secretion of cytokines was measured. OP extracts were also marketed as feed additives, especially fish feed [63], while an olive oil bioactive extract was tested in fish diet, favouring growth of rainbow trout and gilthead sea bream [64]. Other important examples of the use of by-products for functional and food applications have been summarized by Nunes et al. [65] in a more focused review.

As shown in Figure 1, OMWW (also called *alpechin*) is a secondary product of the olive oil extraction process generated from three-phase decanters. It has a distinctive odour, pH = 4.0–5.5, and high electrical conductivity (6000–16000 mS/cm); it also contains large amounts of suspended solids and high concentrations of polyaromatic compounds [66]. Despite the fact that three-phase centrifugation causes the reduction of natural antioxidants in olive oil and a considerable volume of OMWW, it is currently largely used for oil production, having high working capacity and enabling the automation of industrial plants, leading to a lessening of manual labour and olive-processing costs [67]. The physicochemical features of OMWW largely depends on the oil extraction and processing methods, climate, as well as olives maturity, cultivar and origin [68,69,70]. From the chemical composition point of view, OMWW consists mainly of water (80–92%), and contains 3–15% organic matter (olive oils, carbohydrates, lipids, pectin, organic acids, polysaccharides, phenols, tannins, and lignin) and minerals [71]. Phenolic compounds, sugars, and organic acids are the main components of the OMWW organic fraction, while, among minerals, the potassium ion content is relatively high (see Table 1 in Ref. [5]). Long-chain fatty acids contained in OMWW represent a pollution concern, being toxic to soil micro-organisms and plants [68] it is considered the most polluting waste generated by the agri-food industries [71]. Although it has been spread for many years into soil or nearby streams and rivers, OMWW can be very harmful to soil microflora, plants and freshwater species [72] since it is characterized by high values for key pollution parameters, such as biological oxygen demand (BOD_5_, 40–95 g/L) and chemical oxygen demand (COD, 50–180 g/L) and high concentrations of phenols and flavonoids (from 0.5 to 24 g/L), having strong antimicrobial and phytotoxic properties [73]. At present, more than 50 different phenolic compounds, in particular hydrophilic species such as phenolic alcohols and acids and, at in a minor amount, secoiridoids, have been identified in OMWW [74,75,76,77]. Due to its inherent features and with the aim of giving value to a waste, different employments of OMWW have been evaluated in the last 30 years.

The effects of spreading OMWW on soil properties and crops was reviewed by Barbera et al. [78]; within the European Union each State has established different limits (for example, in Italy the legal limit is 80 m^3^/ha) and the authors, by using an holistic approach, concluded that direct application of OMWW exerts a temporary positive effect on soil physical properties, if some outlined restrictions based on soil characteristics are applied. Moreover, they emphasized that polyphenols are the most limiting factor for spreading OMWW on soils because of their antimicrobial and phytotoxic effects [78].

Due to the well-known beneficial effects of phenols contained in OMWW, its potential application for the preparation of functional beverages has been also explored [67]. However, some issues were evidenced, such as the stability of bioactive constituents during preservation procedures, processing, and storage that require appropriate thermal and light-exposure conditions. Moreover, as already discussed, the OMWW composition largely depends on specific factors, which makes a “standardization” of its phenolic extract not straightforward. Olive oils enriched with phenolic compounds extracted from OMWW have been also proposed [79,80,81]; despite further studies are needed, this is an intriguing example of the possibility to create added value by using a waste of olive oil production. As reviewed by Caporaso et al. [81], a similar approach was used for milk and derivatives and also for meat-based products, such as lard and hamburgers.

## 3. Structural Characterization of Phenolic Bioactive Compounds in Waste Products

### 3.1. Olive Leaves

**Figure 3:**
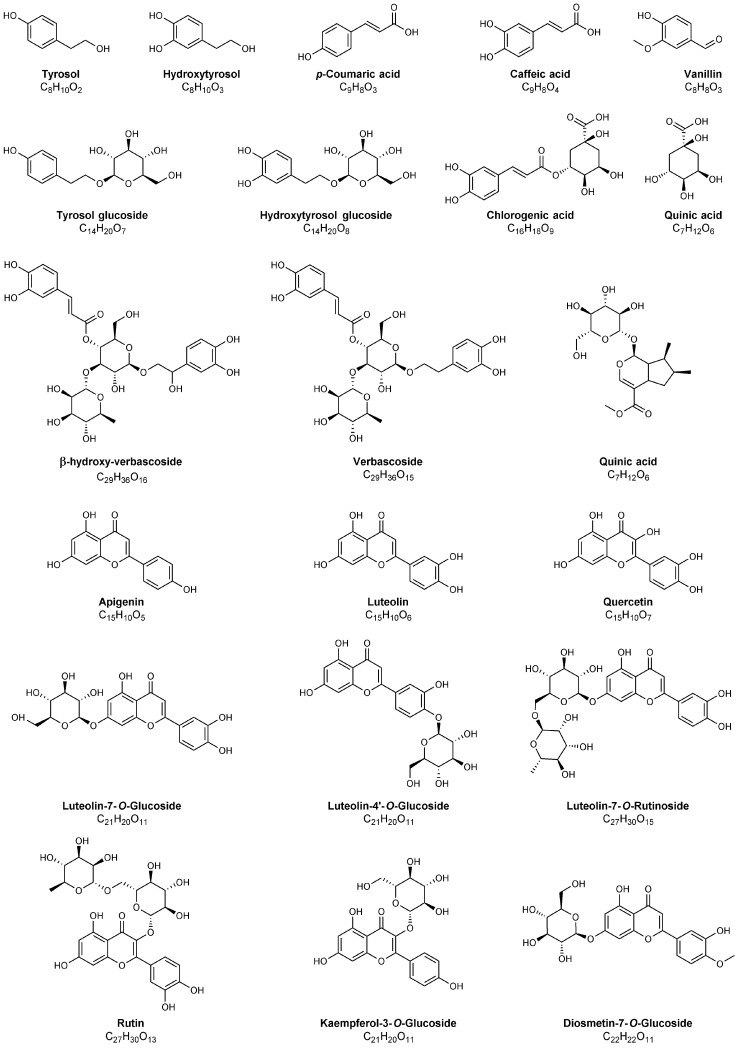
Structures of bioactive compounds, different from secoiridoids, identified in olive leaves, pomace and in olive oil mill wastewater by MS. Compounds belonging to the classes of phenolic glucosides, simple phenols and flavonoids are indicated. For an extended list of bioactive compounds, see Table 2.

The history of studies aiming at the structural characterization of phenolic bioactive compounds in olive leaves is more than 60 years old, with the first structural investigation dating back to 1960, when Panizzi et al. [82] established that the most abundant phenolic compound in olive leaves, initially isolated by Bourquelot and Vintilesco in 1908 and named *oleuropein* [83], was an ester of HT and elenolic acid glucoside; this last is a carboxylic acid belonging to the class of secoiridoids (see Figure 2 and Figure 3). This research came almost a decade after one of the first studies evidencing, on a scientific basis, the positive effects exerted on human health by olive leaves infusions or decoctions [84]. In the 1970s, Inouye et al. used NMR spectroscopy to describe the structures and assign the absolute configurations of oleuropein and of other secoiridoids found in leaves of different plants, namely loganin and nuzhenide (see Figure 2 and Figure 3), opening the way to the first GC-MS systematic investigation on many secoiridoids and iridoid glucosides [85,86,87]. Indeed, 33 secoiridoids and iridoid glucosides present in leaves of *Paederia scandes* and *Lonicera morrowii*, plants belonging to the *Rubiaceae* and *Caprifoliaceae* families, respectively, were characterized. A method for the correct assignment of chromatographic peaks to iridoid/secoiridoid glucosides, was established by the same authors. Among further phenolic compounds existing in olive leaves, also belonging to the class of secoiridoids, ligstroside and demethyloleuropein (with the latter corresponding to oleuropein missing the methyl group linked to the carboxylic moiety bonded to C^4^, see Figure 2) were reported for the first time in 1973 [88], whereas the aglyconic forms of the main secoiridoids of olive leaves were explored by NMR in 1986 by Gariboldi et al. [89]. In the same study, the release of elenolic acid glucoside by acid hydrolysis of oleuropein was proposed, thus confirming the isolation procedure of Panizzi et al. [82]. NMR chemical shifts confirmed a previous spectroscopic investigation dealing with the structure and absolute stereochemistry of elenolic acid [90]. In 1988, Kuwajima et al. [91] isolated oleuroside, another glycosidic secoiridoid from olive leaves; NMR spectroscopy showed that it was an isomer of oleuropein having a 8,10-exocyclic functionality instead of the 8,9 one characteristic of oleuropein and ligstroside (see structures reported in Figure 2 for these secoiridoids with carbon atoms numbering conventionally adopted for them). Notably, the first application of MS was based on fast atom bombardment (FAB), which currently is an unused soft ionization source, to structurally characterize glycosidic secoiridoids as monoprotonated adducts. In subsequent years, FAB-MS using glycerol as matrix was successfully applied to identify the occurrence of oleuropein and its aglycones, obtained by chemical and enzymatic hydrolysis [92], along with flavonoids extracted from olive leaves [40].

To simplify the complexity of phenolic compounds, most of these investigations required several isolation steps from the raw extracts, which may lead to unwanted side reactions of plant tissue metabolites. For this reason, a reliable method for the direct analysis of the olive leaf extract was searched for. In 1997, De Nino et al. [93] set up the first method of direct detection and identification of olive leaves metabolites by MS, with the aim to provide a cultivar “chemical labelling” and a fast non-destructive method to identify biologically and pharmacologically important phenols. Ionspray ionization (ISI, which was the name given at that time to pneumatically assisted electrospray ionization) and FAB were explored to obtain positive ion mass spectra of methanolic extracts of fresh olive foliage. ISI generally led to the generation of protonated molecules, but adducts involving calcium, ammonium or alkali metal ions were also detected, even in the absence of added salts including them as cations. In the case of oleuropein (nominal molecular mass, 540 Da), the ISI mass spectrum displayed peaks at *m/z* ratios 541 (7%), 558 (100%) and 563 (42%), assigned respectively to [M + H]^+^, [M + NH_4_]^+^ and [M + Na]^+^ species. Proton and ammonium adducts were selected as precursor ions for low-energy collisional induced dissociation (CID) tandem mass spectrometry (MS/MS) experiments, leading to similar fragmentation patterns. Intense signals related to ammonium adducts were found in FAB mass spectra of secoiridoid glucosides. Using the same analytical approach, ligstroside and diglycosidic secoiridoids, like nuzhenide (see Figure 2), were also easily identified in the crude extract of olive leaves [93].

The increasing significance of electrospray ionization (ESI) and atmospheric pressure chemical ionization (APCI) as soft ionization sources became the key to couple high performance liquid chromatography (HPLC) and MS with the aim to detect bioactive compounds in olive leaves extracts. Verbascoside, rutin, luteolin-7-O-glucoside, oleuropein and HT (see Figure 2 and Figure 3) were thus detected in olive leaves from three different Portugal cultivars by HPLC-APCI-MS based on an ion trap analyser [94]. Oleuropein and ligstroside, among secoiridoids, and the following compounds: flavonol glycosides (quercetin-3-*O*-rutinoside, quercetin-3,7-*O*-dirhamnoside, kaempferol-3-*O*-glucoside and kaempferol-3-*O*-glucoside-rhamnoside), flavone glycosides (apigenin-7-*O*-rutinoside, apigenin-7-*O*-glucoside, diosmetin-7-*O*-glucoside) and anthocyanins (peonidin-3-*O*-rutinoside, cyanidin-3-*O*-rutinoside), for which representative structures are reported in Figure 3, were identified by LC-ESI-MS based on a quadrupole mass spectrometer in leaves of different olive cultivars harvested in Italy [95]. Interestingly, quantitative estimates of oleuropein content, ranging between 0.13 and 21.34 g/kg of leaves, according to shoot type, tree age, and period of collection were reported for the *Leccino* olive cultivar.

Lujan et al. used LC-ESI-MS/MS relying on a triple quadrupole mass spectrometer to evaluate nutraceuticals extracted from olive-trees [96], and quantitative estimates were reported for some of them, those for which standards were available (see Table 3). Raw extracts of olive leaves were investigated by Goulas et al. [97] for antioxidant potency, screened for antiproliferative activity in endothelial and in two cancer cell lines and analyzed by LC-ESI-MS to identify their major components, namely, HT, luteolin-7-O-glucoside, luteolin-4′-O-glucoside (see Figure 3), oleuropein, luteolin and HT-acetate, that were also quantified (see Table 3). The concomitant variation of olive-leaf phenolic composition during maturation and its influence on the antioxidant capacity was assessed by LC-ESI-MS analyses of young and mature *Olea europaea* L. leaves. Sample extracted by dichloromethane, ethyl acetate and methanol were examined by ESI-MS and MS/MS based on a quadrupole ion trap spectrometer [98]. Apparently, mature olive-leaf extracts contained higher levels of verbascoside isomers and glycosidic forms of luteolin, while higher contents of oleuropein, ligstroside, and flavonoid aglycones were present in young leaves [98]. A qualitative screening of phenolic compounds in olive leaf extracts by HPLC-ESI coupled to time-of-flight MS instrument (TOF-MS) and ion trap tandem mass spectrometry (ESI-IT-MS^2^) was subsequently performed by Fu et al. [99]. In conjunction with LC, the same approaches were exploited to characterize the olive leaf phenolics extracted by microwaves assistance [100]. A similar approach was reported by Quirantes-Piné et al. [101] using a hybrid QTOF-MS instrument with preliminary HPLC separation and an extended investigation on phenolic compounds in leaves of Spanish olive cultivars by LC-ESI-TOF-MS was described by Talhoui et al., with a detailed comparative quantification of several compounds (see Table 3 for the respective values) [102]. Tandem MS was successfully utilized in other studies dealing with the profiling of olive leaves phenolics extracts [103,104,105]. In 2018, Olmo-Garcia et al. studied the distribution of the main secondary metabolites found in *Olea europaea* L., including phenolic compounds, by LC-QTOF-MS (coupled with ESI and/or APCI) and GC-APCI-QTOF-MS [106]. Eight different sample types (olive leaf, stem, seed, fruit skin and pulp, as well as virgin olive oil, olive oil obtained from stoned and dehydrated fruits and olive seed oil) related to the *Picudo* olive cultivar were analyzed. More than 130 metabolites were discovered. The suitability of each platform and polarity (negative/positive) to determine every family of metabolites was evaluated in-depth considering the oil extract from stoned and dehydrated olives, since these matrices contained the major number of compounds. The LC-ESI platform operated in positive ionization mode produced the highest ionization rates for phenolic acids and aldehydes, secoiridoids and derivatives, flavonoids and lignans, therefore indicating LC-ESI(+)-MS as the most efficient choice to ionize these classes of compounds. GC-APCI-MS was the best option for the detection of simple phenols and fatty acids-related metabolites [106].

In recent years, the use of high resolution/accuracy MS, based on the orbital trap FT mass analysers, has opened interesting perspectives for an even deeper structural characterization of bioactive phenolic compounds related to the olive plant. In 2013, Kanakis et al. [107] proposed an HPLC-FTMS study using an Orbitrap analyzer for the identification and quantification of bioactive compounds in olive drupes and olive oils. Michel et al. used the same analytical approach for an extended investigation on olive leaves metabolites, with 86 compounds identified, including coumarin, described for the first time in *O. europaea* [108]. The presence of functional compounds in different Greek varieties of olive leaves and drupes was assessed by Kritikou et al. using reversed-phase LC coupled to high resolution mass spectrometry [109].

More recently, a detailed characterization of the complex set of isomeric forms found for major secoiridoids present in olive oil has been reported by Abbattista et al. using an hybrid quadrupole-Orbitrap mass spectrometer, capable of high collisional energy MS/MS analyses under high resolution/accuracy conditions, and implementing H/D exchange experiments to recognize stable enolic tautomers related to aglyconic secoiridoids [110,111]. The described approach appears very promising for the search for isomeric secoiridoids also in olive leaves extracts.

All above mentioned phenolic compounds, including phenolic acids (like *p*-coumaric and caffeic, see Figure 3), phenolic alcohols (HT and tyrosol), flavonoids (luteolin 7-*O*-glucoside, luteolin 4-*O*-glucoside, rutin, apigenin 7-*O*-glucoside), and oleuropein, among phenolic secoiridoids, represent key components of olive leaves, in accordance with the fact that leaves are important sites of plant primary and secondary metabolism [112]. Many of these compounds play fundamental roles for plant sustenance and defense mechanisms. In particular, oleuropein is one of the most important bioactive compounds present in olive leaves [112], with antioxidative, antimicrobial [113], antiviral (even against the HIV virus [114]), cardioprotective [115], antihypertensive [116] and anti-inflammatory hallmarks [117]. Hypocholesterolemic [118] and hypoglycemic [119] activities, together with the lipid metabolism enhancement effect, potentially useful to alleviate obesity [120], have been also attested in the literature. The role of oleuropein as a mitigating agent for kidney injury induced by the well-known anti-tumoral drug cisplatin, through conjugate generation, has been explored by LC-ESI-MS/MS [121].

As evidenced by data reported in Table 3 and in a further reference [122], oleuropein is, by far, the most abundant bioactive compound in olive leaves, although significant differences can be observed between cultivars. It is, thus, not surprising that the isolation and subsequent chemical characterization of oleuropein by MS has become increasingly important in view of its inclusion in foodstuffs or in other types of products, as explained before. It is worth noting, however, that, although nutraceutical formulations have a very distinctive antioxidant capacity and a greater concentration of oleuropein compared to simple leaf extracts, the effects of the pure substance may be limited by its hydrolytic cleavage during purification steps. The use of olive leaf extracts without isolating the major constituent might thus be recommended to achieve the best healthy properties, thanks to the synergy of all bioactive compounds occurring in the extracts, which most likely affect their absorption and bioavailability [13]. As evidenced in Table 3, other secoiridoids, phenolic or not, like ligstroside, oleuroside, secologanoside, verbascoside, demethyloleuropein and methoxyoleuropein, are usually present in amounts of some g per kg of olive leaves. Notably, secoiridoids like oleuropein stand out among natural compounds synthesized by plants of the Oleaceae family also for their role as precursors of *phytoalexins*, i.e., compounds used as chemical weapons against pathogen attacks, like the corresponding aglycones [123,124,125], thus their isolation might open new interesting perspectives of application.

As for other potentially bioactive compounds for which a quantification has been reported so far in olive leaves, thanks to the availability of the corresponding standards, data reported in Table 3 indicate flavonoids, as such or as glucosides, especially luteolin-4′-*O*-glucoside, to be generally present in amounts of several g per kg of olive leaves, that would encourage the possibility of extracting them for subsequent applications.

### 3.2. Olive Pomace (OP) and Olive Oil Mill Wastewater (OOMW)

By analogy with olive leaves, bioactive phenolic compounds can occur in OP [126]. A summary of the most important bioactive compounds identified so far in olive leaves, OP and OOMW using MS techniques is reported in Table 2.

Although the classical Folin–Ciocalteu method is often applied to assess the total content of phenolic compounds also in plant-related extracts [141], analytical approaches enabling the characterization of specific compounds are clearly required to assess the possibility to recover valuable components [142]. In this context, RP, hydrophilic interaction liquid chromatography (HILIC) and supercritical fluid chromatography (SFC) have been employed as analytical separating means of phenolic compounds in OP [49]. While the use of UV–Vis DAD or fluorescence detectors (FD) has been reported [50], ESI-MS and, recently, MALDI-MS [76], are most useful to provide detailed structural characterization. As an example, ultra-high performance liquid chromatography (UHPLC) and high resolution MS led to overcome the problems due to the co-elution of HT-4-*β*-D-glucoside and HT occurring in the olive pulp, OMWW, and OP extracts [143]. The existence of flavonol glucosides, anthocyanins and derivatives of hydroxycinnamic acids in olive oil processing wastes can be easily predicted, since such compounds were reported to be present in olive drupes already in studies dating back to two decades ago [144,145]. Cardoso et al. [130,146] compared the methanol extract of olive pulp and OP and collected 27 fractions that were further investigated by ESI-MS. As a result, some common phenolic compounds, many of which found also in olive leaves, namely, verbascoside, rutin, caffeoyl-quinic acid, luteolin-4-glucoside, 11-methyl-oleoside, HT-1′-*β*-glucoside, luteolin-7-*O*-rutinoside, oleoside (with two derivatives, 6′-*β*-glucopyranosyl-oleoside and 6′-*β*-rhamnopyranosyl-oleoside), and 10-hydroxy-oleuropein were found. An oleuropein glucoside previously detected in olive leaves was also found in both matrices. In quantitative terms, most of the phenolic compounds were found in equivalent amounts in olive pulp and pomace, except oleoside, which was the main phenolic compound in olive pulp (31.6 mg/g) but was tenfold lowered in OP, and oleuropein, almost absent in OP (2.7 mg/g in the pulp). Cioffi et al. [147] used HPLC-DAD analysis to assess the phenolics content in virgin olive oils and OP of samples collected in the area of Cilento (Campania, Southern Italy). In all the investigated samples, secoiridoids represented about 50–70% of the total phenolic compounds with oleuropein and ligstroside aglycone being the most abundant ones, although oleocanthal (see Figure 2) was also identified and quantified. Among simple phenols, gallic acid, HT, and tyrosol were the major constituents in olive oil, while caffeic acid, tyrosol, and gallic acid prevailed in OP (see Table 3). Moreover, leaves and olive extracts were found to elicit a strong free-radical scavenging activity, assessed using the 2,2-diphenyl-1-picryl-hydrazyl-hydrate (DPPH) test and correlated to the high total content of phenols, while the OP extract showed a lower antioxidant activity. In another study [148], the concentration of oleocanthal was evaluated both in OP and freshly pressed extra-virgin olive oils (EVOO) obtained after early, mid and late season harvests. While similar levels of oleocanthal were found in pomace and in EVOO from early and mid harvests, the EVOO obtained after late harvest showed greater contents compared to pomace, likely due to chemical and enzymatic degradation of olive drupes with maturation. These findings suggested that pomace waste from early and mid harvests might represent a precious source of oleocanthal. Lozano-Sanchez et al. [149] characterized phenolic compounds and polar molecules occurring in both solid and aqueous wastes arising from olive oil production by LC coupled to ESI-TOF-MS and IT-MS. Phenolic compounds such as vanillin, HT, tyrosol, luteolin, apigenin and quinic acid (see Figure 3 and Table 3) were quantitated using external calibrations of commercial standards. When no commercial standard was available, the quantitation was accomplished by compounds with similar chemical structures. Therefore, secoiridoids and lignans were respectively quantified using oleuropein and (+)-pinoresinol standards. The main components of phenolic fraction in solid wastes were found to be the dialdehydic form of decarboxymethyl-elenolic acid (see Figure 2) and HT, with concentrations ranging from 153 to 601 mg/kg and from 159 to 181 mg/kg, respectively. Among secoiridoids, oleuropein aglycone and its hydroxylated and decarboxymethylated derivatives were the most abundant compounds. The same authors proposed the occurrence of novel oxidized and hydrated compounds related to elenolic acid.

The selection of the extraction solvents must be carefully made not only considering the chemical and physical properties of the target substances while minimizing matrix interferences but also avoiding toxic, inflammable, and hazardous mixtures. Lunque de Castro and coworkers [127] reported a detailed investigation of phenolics and other polar compounds occurring in OP extracted by a superheated water/ethanol mixture 50:50 (*v*/*v*) at 160 °C. The identification was validated by accurate *m*/*z* ratios and isotopic patterns of precursor ions and their related product ions. In a subsequent paper, minor secoiridoids and other phenolic compounds were identified in the *alperujo* extract, treated with polyamide and XAD resin, using ESI-FTMS [128]. The extract was concentrated and purified by solid phase extraction (SPE) using a RP C18 cartridge, collecting three main fractions enriched in minor compounds. The latter corresponded to several oleuropein derivatives, as oleoside methyl ester, dihydro-oleuropein, neo-nüzhenide (an analogue of nüzhenide including HT, instead of tyrosol) and oleuropein diglycoside, never identified before. A novel secoiridoid glucoside, i.e., 1-*β*-D-glucopyranosyl acyclodihydroelenolic acid, was recognised in *alperujo* for the first time also recurring to NMR [128]. Especially during the first decade of the new millennium, this last spectroscopic technique has played an important role in the identification of phenolic compounds in OP and OMWW, by analogy with olive leaves. As an example, the purity of 3,4-dihydroxyphenylglycol (DHPG), a compound corresponding to HT having an additional OH group on the ethyl chain, described as a strong antioxidant component of both OP and OMWW, was assessed by NMR [152]. In a subsequent paper, the precursors of DHPG were identified and characterized after extraction from *alperujo*; three compounds, all containing a DHPG moiety, were recognized as precursors, namely the diastereoisomeric forms of both *β*-hydroxy derivatives of verbascoside and isoverbascoside (*β*-hydroxyacteoside and *β*-hydroxyisoacteoside), and 2″-hydroxyoleuropein [129].

By combining ^1^H NMR, ^13^C NMR, IR, UV and ESI-MS Rigane et al. [137] identified a new iridoid compound, deoxyloganic acid lauryl ester, in OP obtained from a two-phase decanter during the extraction of *Chemlali* olive oil; the antioxidant activity of the pure compound was assessed by in vitro tests. Damak et al. [140] reported the first isolation and characterization of Olenoside A and its known epimer Olenoside B, present in OMWW as an isomeric mixture. Their structures were determined by 2D NMR and confirmed, for the most abundant isomer, by X-ray diffraction. Further, 1D and 2D NMR spectroscopy was proved to be a useful tool for olive oil production wastes fingerprinting without separation and pre-treatment of samples. The chemical composition of major and minor components, like secoiridoids, lignans, and phenolic compounds present in chloroform extracts of olive paste and pomace was evaluated by this approach [153]. Rapid NMR fingerprinting can be used to determine the entire profile of phenolics in different waste materials generated by the olive oil production process, thus contributing to their valorisation. For example, Hafidi et al. studied the aerobic digestion of OMWW under various medium conditions to determine the best treatment of residue stabilisation; FTIR and ^13^C NMR spectroscopies were used to compare results showing that treatment of OMWW by soil micro-flora with pH neutralization by phosphate could be considered the best one, allowing good stabilisation of organic matter and high preservation of nitrogen in the humic form [154]. More recently, the degradation of OMWW by different methods was studied by FTIR: results showed that treatment with H_2_O_2_ under high pressure and temperature removed 89.2% of COD, 91.5% of phenolic compounds, and the colour of OMWW; the comparison of NMR spectra confirmed that all phenolic compounds and the majority of other compounds were abated [155].

Turning back to phenolic compounds and their investigation by MS, a direct and fast analytical method based on UHPLC-DAD coupled with ESI/MS-MS was applied to OP by Malapert et al. [49]. In a short analysis time (ca. 12 min), thirty-five metabolites belonging to the classes of phenyl alcohols, secoiridoids, flavonoids, and iridoids, including a novel *p*-coumaroyl aldarate, a verbascoside derivative and a new ligstroside derivative, were identified as the main constituents of *alperujo*. More recently, an extended overview of the distribution of bioactive compounds along the entire production chain, from drupes to oil and processing wastes was described by Russo et al. [151] by LC-MS.

As recently evidenced for olive oil secoiridoids, whose profile is affected by various parameters, including olive cultivar and geographical origin [156], ripening stage at the harvesting time, agronomic and technological practices [157,158], and whose integrity was influenced by storage conditions [158], the distribution and integrity of phenolic compounds contained in OP and OOMW is expected to be affected as well. These differences are manifest when a comparison in quantitative terms is attempted (see Table 3). Among them, storage is particularly challenging since those by-products of olive oil production can be stored in ponds even for some months during the olive oil processing season. Brenes et al. [159] compared the phenolic content of refined oils as lamp oil, crude OP oil and second centrifugation olive oils at various storage times, observing an increase of HT, HT-acetate, tyrosol, catechol, flavonoids luteolin and apigenin and vanillic acid with storage time. HPLC-MS and GC-MS were employed to identify 4-ethylphenol, responsible for the unpleasant odour of “wet horse”, which was also intensified with time storage. Medina et al. [60] studied the changes of phenols during both the storage phase in open air ponds and the extracting process. Considering the decrease of oleuropein and HT-glycoside concentrations from harvested olives to *alperujo* they concluded that most of this *o*-diphenolic species were lost mainly due to enzymatic oxidation during malaxation and further manipulation steps of the olive paste. The technique adopted for the extraction from OP can also create different qualitative and quantitative phenolic profiles. Using pressurized liquid extraction under optimized conditions, the total amount of secoiridoids and flavonoids was three- and four-times higher than that found in conventional extracts, as determined by HPLC-DAD-ESI-TOF/MS [132]. In a further study, supercritical carbon dioxide was used to extract oil from *alperujo* and the total phenol content was very high as assessed by HPLC-DAD, with the highest HT content (1.9 g/kg) being recovered [160].

Even if a certain degree of variability exists, it can be resumed that the most abundant phenolic compounds in OP are HT (1.8%), tyrosol, *p*-coumaric acid, oleuropein, vanillic acid, verbascoside, cinnamic acid, caffeic acid, elenolic acid, catechol, ferulic acid, gallic acid, syringic acid, sinapic acid, HT-10-*β*-glucoside, homovanillic acid, demethyloleuropein, ligstroside, 11-methyloleoside. Among flavonoids, luteolin, luteolin 7-*O*-glucoside, luteolin-4-*O*-glucoside, luteolin-7-*O*-rutinoside, rutin, hesperidin, quercetin, apigenin, apigenin 7-*O*-glucoside, cyanidin 3-*O*-rutinoside, and cyanidin 3-*O*-glucoside can be considered the most relevant [120,161] (See Table 2 and Table 3). Since the amount of several of these compounds in OP can often exceed that found in olive oils, their recovery may represent a convenient strategy for their use in the food, cosmetic and pharmaceutical industries. However, as highlighted from quantitative data, the retrieved amount largely depends on several factors which makes necessary a standardization of the phenolic extraction.

OMWW is an important by-product of olive oil production in which a relevant fraction of hydrophilic bioactive compounds is expected to be transferred into it. One of the first LC-ESI-MS/MS method of tyrosol and HT quantification in OMWW was proposed by Bazoti et al. in 2006 [139]. Specific transitions were observed in negative ion mode, i.e., 137→119 for tyrosol, 153→123 for HT and 201→125 due to 2-(5-ethylidene-2-oxo-tetrahydro-2H-pyran-4-yl) acetic acid, which is generated by cleavage of oleuropein aglycone. Good linearity, precision, and accuracy was demonstrated because of baseline resolution of chromatographic peaks, combined with the MS selectivity of multiple reaction monitoring.

The profile of elenolic acid derivatives in OMWW was very recently proposed by Mattonai et al. [138] using a LC column with embedded polar groups. HT and tyrosol were found as main components (see Table 3) and an important depletion in the phenolic content of OMWW after one year of storage was proved. Moreover, unknown components, such as HT-esters of elenolic acid were identified and their presence was evidenced as a marker for evaluating the OMWW ageing. UPLC-ESI-MS/MS was used by Suàrez et al. [131] with the aim of preparing phenolic extracts from olive cake for potential application as food antioxidants; a complete list of phenolic compounds obtained from the solid and the liquid fraction of olive cake was reported. An interesting demonstration of the high variability of OMWW composition is represented by the study of Cardoso et al. [162], who found seven new bioactive phenols in two Portuguese OMWW using ESI-MS and MS^n^ analyses. Oleuropein and ligstroside isomers including the glucose unit linked to a phenolic OH group were discovered. Moreover, ions detected at nominal *m/z* ratios 863, 685 and 847 were respectively assigned to glucosidic derivatives of the oleuropein isomer and a mono- and di-glucosides of the ligstroside isomer, thus showing that even secoiridoids with a relatively high molecular weight can be found in OMWW. Similarly, verbascoside, isoverbascoside and their derivatives were characterized and quantified via LC-DAD-MS/MS analysis by Cardinali et al. [135] employing low-pressure gel filtration chromatography. The potentiality of RPLC-ESI-DAD-MS for olive mill waste characterization was confirmed by Obied et al. [134], that demonstrated the presence of 52 compounds in OMW; with a similar approach, 23 compounds, belonging to secoiridoids and their derivatives, phenyl alcohols, phenolic acid and derivatives, and flavonoids, were identified in OMWW coming from Italian and Greek olive cultivars by D’Antuono et al. [136]. In quantitative terms, OMWW seems a very promising source of valuable compounds as oleacin, tyrosol, HT, verbascoside, HT glucoside, caffeic acid, gallic acid, luteolin, oleoside, oleuropein and derivative, oleuroside and rutin (see Table 3).

Among MS-based techniques applied to the characterization of phenolic compounds in olive oil production by-products, specific considerations are deserved by MS relying on matrix assisted laser desorption ionization (MALDI). Despite the well-known advantages of MALDI, such as rapidity, sensitivity, easy sample preparation, MALDI-ToF/ToF MS has rarely been applied to the analysis of secoiridoids and closely related by-products. This task has been recently accomplished in our laboratory by using binary matrices such as 1,8-bis(dimethylamino) naphthalene/9-aminoacridine (DMAN/9AA) [163] or 1,8-bis(tetramethylguanidino)naphthalene/9-aminoacridine (TGMN/9AA) in negative ion mode [76]. As a result, HT and decarboxymethyl-elenolic acid were identified as major component; secoiridoids oleuropein aglycone and oleacein were detected in the MALDI-MS spectrum of OMWW as well. Moreover, the identification of 3,4-dihydroxyphenyglycol, the geminal diol of oleacein and an hydroxylated derivative of decarboxymethyl-elenolic acid was tentatively made [76]. The same approach was also applied to olive leaves extracts and oleuropein was confirmed as the major phenolic compound. Luteoline, oleoside, secologanoside (see Figure 2) and isomeric oleuropein diglucosides were also identified.

## 4. Conclusions

The application of increasingly advanced mass spectrometry techniques over almost three decades, boosted by the introduction of soft ionization sources and, recently, also MALDI, has led to a remarkable amount of information on potentially bioactive compounds, mainly belonging to the classes of phenolics and secoiridoids, in typical by-products of olive oil production, namely olive leaves, olive pomace and olive oil mill wastewater. Liquid chromatography has certainly played a key role, enabling the preliminary separation of several compounds in such a complex type of matrices. With the increasing awareness of the potential of these by-products as sources of valuable compounds, useful for different types of industries (food, cosmetics, etc.), the role of mass spectrometry in estimating which of them is more promising in the perspective of recovering a specific compound is expected to become progressively more relevant in the next future.

## Figures and Tables

**Table 1:** Main uses of olive leaves in traditional medicine occurring worldwide.

Country	Assumption Form of Olive Leaf-Based Products	Route	Treatments	References
Arabic countries	Dried plant fumigation	Nasal	Abortifacient, and treatment of cystitis and sore throat	[35]
Brazil	Herbal tea of the fresh leaves	Oral	To induce diuresis, and treatment of hypertension	[36]
Canary Islands	An infusion prepared from the fresh or dried leaves	Oral/rectal	Treatment of diabetes; hypertension and haemorrhoids	[37]
France	Powdered dried leaves as hard capsules	Oral	To promote urinary and digestive elimination functions	[38]
Germany	Extract with ethanol 96% *v*/*v* as liquid or coated tablet	Oral	Treatment of atherosclerosis and hypertension	[38]
Italy	Infusion of the dried leaf Infusion of the fresh leaf	Oral	Treatment of hypertension and anti-inflammatory; for wound healing, emollient for ingrown nails and to restore epithelium	[39,40]
Morocco	Leaves and essential oil from the leaves	Oral Topical	Treatment of stomach and intestinal disease and as a mouth cleanser; treatment of constipation and liver pain; tonic for hairs	[41]
Trinidad	Hot water extract of the leaf	Oral	To increase milk supply of nursing mother	[42]
Ukraine	Hot water extract of dried plant	Oral	Treatment of bronchial asthma	[43]

**Table 2:** List of the most important bioactive compounds detected using mass spectrometry in olive leaves and olive pomace and in olive oil mill wastewater (OOMW).

Name	Formula (M)	*m/z* [M-H]^-^	*m/z* [M + H]^+^	Ionization Source	Leaves	Olive Pomace	OOMW
10-hydroxy-oleacin	C_17_H_20_O_7_	335.114	337.128	APCI, ESI	[106] ^5^	[49]	
10-hydroxy-oleuropein	C_25_H_32_O_14_	555.172		ESI	[100]	[127]	
10-hydroxyoleuropein aglycone	C_19_H_22_O_9_	393.120	395.133	APCI, ESI	[106] ^5^		
1-*β*-D-glucopyranosyl acyclodihydroelenolic acid glycosylate	C_23_H_28_O_16_	569.209		ESI		[128]	
1-*β*-D-glucopyranosylacyclodihydro elenolic acid (acyclodihydroelenolic acid hexoside)	C_17_H_28_O_11_	407.156	409.17	APCI, ESI	[106] ^5^	[128]	
2-(2-ethyl-3-hydroxy-6-propionylcyclohexyl) acetic acid glucoside	C_19_H_32_O_9_	403.197		ESI	[100]		
2-hydroxy-oleuropein	C_25_H_32_O_14_	555.172		ESI		[129]	
2-phenethyl-*β*-primeveroside	C_19_H_28_O_10_	415.161	417.175	APCI, ESI	[106] ^5^		
3,4-dihydroxyphenylglycol	C_8_H_10_O_4_	151.040 ^d^	153.054 ^a^	APCI, ESI, MALDI	[106] ^5^		[76] ^1^
4-hydroxyphenylacetic acid	C_8_H_8_O_3_	151.040	153.054	APCI, ESI	[106]^5^	[130]	
6′-*β*-rhamnopyranosyl-oleoside	C_22_H_32_O_15_	535.167		ESI		[130]	
6′-O-[2,6-dimethyl-8-hydroxy-2-octenoyloxy]-secologanoside	C_26_H_38_O_13_	557.224		ESI	[101]		
7-deoxyloganic acid	C_16_H_24_O_9_	359.135		ESI	[108]	[127]	
Acteoside	C_29_H_36_O_15_	623.198		ESI	[103]		
Acetoxypinoresinol	C_22_H_24_O_8_	415.139		ESI	[100]	[131]	
Aesculetin (Dihydroxhycoumarin isomers)	C_9_H_6_O_4_	177.019	179.034	APCI, ESI	[106] ^5^ [108]		
Aesculin	C_15_H_16_O_9_	339.072	341.088	APCI, ESI	[106] ^5^		
Apigenin	C_15_H_10_O_5_	269.046	271.06	APCI, ESI	[9] ^3^ [100] [106] ^5^ [108,109]	[96,127,131,132,133]	[133]
Apigenin 6,8-di-*C*-glucoside (Vicenin 2)	C_27_H_30_O_15_	593.151		ESI	[7]		
Apigenin-7-*O*-glucoside	C_21_H_20_O_10_	431.098	433.113	APCI, ESI, FAB	[7] [9] ^3^ [99,100,101,105] [106] ^5^ [108]	[96,127,131,133]	[133]
Apigenin-7-*O*-rutinoside	C_27_H_30_O_14_	577.156	579.171	APCI, ESI	[7,23,100,101,102] [106] ^5^ [108]		[134]
Ascorbyl hexoside	C_12_H_18_O_11_	337.078		ESI	[7]		
Azelaic acid	C_9_H_16_O_4_	187.097	189.113	APCI, ESI	[106] ^5^		
*β*-hydroxy-isoverbascoside	C_29_H_36_O_16_	639.193		ESI	[7,108]	[128,129]	[135,136]
*β*-hydroxyacteoside	C_29_H_36_O_16_	639.193		ESI		[129]	
*β*-methoxyl-verbascoside	C_30_H_38_O_16_	653.209		ESI	[108]		
Caffeic acid	C_9_H_8_O_4_	179.035	181.05	APCI, ESI	[103,105] [106] ^5^	[49,127,131]	[134,136]
Caffeic acid derivative	C_18_H_18_O_9_	377.089		ESI	[103]		
Caffeic acid diglycoside	C_21_H_30_O_13_	489.161		ESI	[108]		
Caffeic alcohol derivative	C_21_H_30_O_13_	489.161		ESI	[103]		
Caffeoyl-6′-secologanoside (Cafselogoside)	C_25_H_28_O_14_	551.141	553.155	APCI, ESI	[106] ^5^	[49,50,133]	[133,134]
Caffeoyl-hexoside	C_15_H_18_O_9_	341.088		ESI		[49]	
Calceolarioside	C_23_H_26_O_11_	477.14		ESI	[7,108]	[49]	
Chlorogenic acid (Caffeoyl-quinic acid)	C_16_H_18_O_9_	353.088	355.102	ESI	[7,105]	[49,130]	[134]
Chrysoeriol	C_16_H_12_O_6_	299.056	301.071	ESI			[134]
Chrysoeriol-7-*O*-glucoside	C_22_H_22_O_11_	461.109	463.124	APCI, ESI	[7,99,100,102] [106] ^5^		
Cinammic acid	C_9_H_8_O_2_	147.045		ESI		[127]	
Citric acid	C_6_H_8_O_7_	191.02	175.024 ^a^	APCI, ESI	[7] [106] ^5^		
Comselogoside	C_25_H_27_O_13_	535.146	537.16	APCI, ESI	[106] ^5^	[49,50,133]	[133,134,136]
Cyanidin-3-rutinoside	C_27_H_31_O_15_^+^		595.166	ESI	[95]		
D(+)-Erytro-1-(4-hydroxy-3-methoxy)-phenyl-1,2,3-propantriol	C_10_H_14_O_5_	213.077	215.091	ESI		[50]	[134]
Decaffeoyl verbascoside	C_20_H_30_O_12_	461.166		ESI	[7]	[49]	
Dehydro ligstroside aglycone	C_19_H_20_O_7_	359.114	361.129	APCI, ESI	[106] ^5^		
Dehydro oleuropein aglycone	C_19_H_20_O_8_	375.109	377.124	APCI, ESI	[106] ^5^		
Demethyloleuropein	C_24_H_30_O_13_	525.161		ESI	[7,99,102,108]	[132,133]	[133]
Deoxyloganic acid lauryl ester	C_23_H_38_O_5_	393.264		ESI		[137]	
Desoxyelenolic acid	C_11_H_14_O_5_	225.077	227.091	APCI, ESI	[106] ^5^		
Dihydro-oleuropein	C_25_H_36_O_13_	543.208		APCI, ESI	[7] [106] ^5^	[49,127,128,133]	[133]
Dihyroxybenzoic acid hexoside	C_13_H_16_O_9_	315.072		ESI	[7]		
Dihyroxybenzoic acid hexoside pentoside	C_18_H_24_O_13_	447.114		ESI	[7]		
Dimethyl oleuropein aglycone	C_21_H_26_O_8_	405.156	407.17	APCI, ESI	[106] ^5^		
Diosmetin	C_16_H_12_O_6_	299.056	301.071	APCI, ESI	[98,100] [106] ^5^ [108]	[127]	
Diosmetin glucoside	C_22_H_22_O_11_	461.109		ESI	[108]		
Diosmin	C_28_H_32_O_15_	607.167		ESI	[7,100,108]		
Elenolic acid (EA)-methyl ester	C_12_H_16_O_6_	255.088	257.102	APCI, ESI	[106] ^5^		
Elenolic acid (EA)	C_11_H_14_O_6_	241.072	243.086, 225.077 ^a^	APCI, ESI, MALDI	[76] ^1^ [106] ^5^	[49,127,131]	[134,136,138]
Elenolic acid (EA) derivative (decarboxylated form of hydroxyelenolic acid)	C_10_H_14_O_5_	213.077	197.081 ^a^	APCI, ESI, MALDI	[106] ^5^	[132]	[76] ^1^ [138]
Elenolic acid (EA) derivative	C_10_H_16_O_5_	215.092		ESI		[50,132]	
Elenolic acid decarboxymethylated (EDA)	C_9_H_12_O_4_	183.066	185.081	APCI, ESI, MALDI	[106] ^5^	[50]	[76] ^1^ [136,138]
Elenolic acid diglucoside	C_23_H_33_O_16_	565.177		ESI	[101]		
Elenolic acid glucoside (Oleoside methyl ester)	C_17_H_24_O_11_	403.125	405.139	APCI, ESI, MALDI	[7] [76] ^1^ [99,100,101,102,103] [106] ^5^ [108]	[127,128,130,133]	[133,134]
Elenolic acid hexoside derivative	C_19_H_26_O_13_	461.13		ESI	[7]		
Eriodictyol	C_15_H_12_O_6_	287.056		ESI	[103,105]		
Eudesmic acid	C_10_H_12_O_5_	211.06	213.076	APCI, ESI	[106] ^5^		
Ferulic acid	C_10_H_10_O_4_	193.051	195.066	APCI, ESI	[103] [106] ^5^ [109]	[49,127]	
Feruloyl-hexoside	C_16_H_20_O_9_	401.109		ESI		[49]	
Flavonol diglicoside	C_27_H_30_O_16_	609.146		ESI	[108]		
Fraxamoside	C_25_H_30_O_13_	537.161		ESI	[7,108]		
Fustin	C_15_H_12_O_6_	287.056		ESI	[103]		
Gallic acid	C_7_H_6_O_5_	169.014		APCI, ESI	[7,105] [106] ^5^ [109]	[127]	[138]
Gallocatechin	C_15_H_14_O_7_	305.067		APCI, ESI	[106] ^5^ [108]		
Gingerol	C_17_H_26_O_4_	293.176	295.19	APCI, ESI	[106] ^5^		
Gluconic acid	C_6_H_11_O_7_	195.051		ESI	[101]		
Homovanillyl alcohol	C_9_H_12_O_3_	167.071		ESI	[103]		
Hydrogenated EDA	C_9_H_14_O_4_	185.082		MALDI			[76] ^1^
Hydrogenated-EA	C_11_H_16_O_6_	243.087		ESI		[50]	
Hydro-oleuropein	C_25_H_34_O_13_	541.193		ESI		[128]	
Hydroxy-oleocanthal	C_17_H_20_O_6_	319.119	321.134	APCI, ESI	[106] ^5^		
Hydroxy-EA	C_11_H_14_O_7_	257.067	259.081	APCI, ESI	[106] ^5^		
Hydroxy-EDA	C_9_H_12_O_5_	199.062	183.065 ^a^	APCI, ESI	[106] ^5^	[49,132]	[136]
Hydroxy-oleuropein	C_25_H_32_O_14_	555.172		APCI, ESI	[7,101,103] [106] ^5^ [108]	[132]	
Hydroxy-oleuropein aglycone	C_22_H_32_O_8_	423.202		ESI	[108]		
Hydroxypinoresinol	C_20_H_22_O_7_	373.129	375.145	APCI, ESI	[106] ^5^	[127]	
Hydroxytyrosol (3,4-DHPEA)	C_8_H_10_O_3_	153.056	155.07	APCI, ESI, MALDI	[94,97,99,100,101,103] [106] ^5^ [108,109]	[49,50,96,127,131,132,133]	[76] ^1^ [133,134,136,138,139]
Hydroxytyrosol acetate	C_10_H_11_O_4_	195.066		ESI	[97,101]	[131,133]	[133]
Hydroxytyrosol diglucoside	C_20_H_28_O_13_	475.146		ESI		[127]	
Hydroxytyrosol glucoside	C_14_H_20_O_8_	315.109	317.124	APCI, ESI, MALDI	[7,23] [76] ^1^ [99,102,103,106] ^5^ [108]	[49,127,130,133]	[133,134,136]
Hydroxytyrosol rhamnoside	C_20_H_34013_	481.193		ESI		[127]	
Hydroxytyrosil acyclodihydroelenolate	C_19_H_26_O_8_	381.156	383.17	ESI			[134,136]
Isoacetoside	C_29_H_36_O_15_	623.198	625.213	ESI	[99]		[134]
Isoverbascoside	C_29_H_36_O_15_	623.198		ESI	[7]		[135]
Jaspolyoside	C_42_H_54_O_23_	925.298		ESI	[7,108]		
Jaspolyoside derivative	C_42_H_54_O_22_	909.303		ESI	[108]		
Ligstroside	C_25_H_32_O_12_	523.128	525.197 507.185 ^a^ 542.223 ^b^	APCI, ESI, FAB	[7,23] [93] ^4^ [98,99,101,102,103] [106] ^5^ [108]	[132,133]	[133,134]
Ligstroside aglycone (*p*-HPEA-EA)	C_19_H_22_O_7_	361.129	363.144	APCI, ESI	[94,99] [106] ^5^	[127,128,131,133]	[133,134]
Loganic acid	C_16_H_24_O_10_	375.130		ESI	[7]	[127,132]	
Loganic acid glucoside	C_22_H_33_O_16_	537.183		ESI	[7]	[49,127]	
Loganin (7-epiloganin)	C_17_H_26_O_10_	389.145		ESI	[7,101,108]	[127]	
Loganin glucoside	C_23_H_38_O_16_	569.209		ESI		[127]	
Lucidumoside C	C_27_H_35_O_14_	583.203		APCI, ESI	[101] [106] ^5^		
Luteolin derivative	C_31_H_36_O_13_	615.208		ESI	[108]		
Luteolin	C_15_H_10_O_6_	285.041	287.055	APCI, ESI	[9] ^3^ [23,97,99,100,101,102,103,105] [106] ^5^ [108,109]	[96,127,130,131,132,133]	[133,134]
Luteolin derivative	C_31_H_28_O_14_	623.141		ESI	[108]		
Luteolin diglucoside	C_27_H_30_O_16_	609.146	611.161	APCI, ESI	[7,23,100,101,102] [106] ^5^		
Luteolin-4′-*O*-glucoside	C_21_H_20_O_11_	447.093	449.108	APCI, ESI	[94,97,101] [106] ^5^	[130,133]	[133,134]
Luteolin-7-*O*-glucoside	C_21_H_20_O_11_	447.093	449.108	APCI, ESI, MALDI	[4,7] [9] ^3^ [76] ^1^ [94,97,98,101,105] [106] ^5^	[96,128,130,131,132,133]	[133,134]
Luteolin-7-*O*-rutinoside	C_27_H_30_O_15_	593.151	595.166	ESI APCI	[7] [9] ^3^ [101]	[130,132]	[134]
Luteolin-hexoside	C_21_H_20_O_11_	447.093	449.108	APCI, ESI	[7,23,99,100,101,102,103] [106] ^5^ [108]	[127,132]	
Luteolin-*O*-rutinoside	C_27_H_30_O_15_	593.151	595.166	APCI, ESI	[23,100,102] [106] ^5^ [108]	[49,130,133]	[133]
Malic acid	C_4_H_6_O_5_	133.014		ESI	[7]		
Maslinic acid	C_30_H_48_O_4_	471.348		ESI	[108]		
Methoxyloleuropein	C_26_H_34_O_14_	569.188		ESI	[100,102,103,108]		
Methoxyloleuroside	C_26_H_34_O_14_	569.188		ESI	[108]		
Methyl oleacein	C_18_H_22_O_6_	333.134	317.139 ^a^	APCI, ESI	[106] ^5^		
Methyl oleuropein aglycone	C_20_H_24_O_8_	391.14	393.154	APCI, ESI	[106] ^5^		
Monohydrated oleacein (geminal diol)	C_17_H_22_O_7_	337.129		MALDI			[76] ^1^
Monohydrated-EDA	C_9_H_14_O_5_	201.077		MALDI			[76] ^1^
Naringenin	C_15_H_12_O_5_	271.089	273.076	APCI, ESI	[106] ^5^	[132]	
Neo-nüzhenide	C_31_H_42_O_18_	701.23		ESI, MALDI	[76] ^1^ [108]	[128]	
Nüzhenide	C_31_H_42_O_17_	685.235	687.25	APCI, ESI	[99,103] [106] ^5^	[49,133]	[133,134]
Oleacin (3,4-DHPEA-EDA)	C_17_H_20_O_6_	319.119	321.133	APCI, ESI, MALDI	[106] ^5^ [108]	[127,131,133]	[76] ^1^ [133,134]
Oleanolic acid	C_30_H_48_O_3_	455.353		ESI	[108]		
Olenoside A and B	C_11_H_14_O_5_		249.074 ^c^	MALDI			[140] ^1^
Oleocanthal (*p*-HPEA-EDA)	C_17_H_20_O_5_	303.124	305.138	APCI, ESI	[106] ^5^	[127,131,133]	[133,134]
Oleoside	C_16_H_22_O_11_	389.109	391.117	APCI, ESI	[7,23,94,99,101,102,103] [106] ^5^ [108]	[49,50,127,130,132,133]	[133,134,136]
Oleoside diglucoside	C_28_H_42_O_21_	713.215		ESI		[50,127]	
Oleoside deoxyriboside	C_20_H_26_O_15_	505.12		ESI		[49,50,127]	
Oleoside dimethyl ester	C_18_H_26_O_11_	417.14		ESI		[127]	
Oleoside glucoside (6′-*β*-glucopyranosyl-oleoside)	C_22_H_32_O_16_	551.162		ESI		[127,130]	
Oleoside methyl ester derivative	C_18_H_30_O_11_	421.172		ESI	[7]		
Oleuricine A (oleuropein glucoside)	C_31_H_42_O_18_	701.23		MALDI	[76] ^1^		
Oleuricine B (oleuroside glucoside)	C_31_H_42_O_18_	701.23		MALDI	[76] ^1^		
Oleuropein	C_25_H_32_O_13_	539.177	541.193 558.218 ^b^ 523.176 ^a^	APCI, ESI, FAB, MALDI	[7,23] [76] ^1^ [92] ^2^ [93] ^4^ [94,97,98,99,100,101,102,103] [106] ^5^ [108,109]	[96,127,128,130,131,132,133]	[133,134,136]
Oleuropein aglycone (3,4-DHPEA-EA)	C_19_H_22_O_8_	377.124	379.139	APCI, ESI, FAB, MALDI	[23] [76] ^1^ [92] ^2^ [94,99,102] [106] ^5^ [108]	[49,127,131,133]	[76] ^1^ [133,134]
Oleuropein aglycone derivative	C_19_H_20_O_7_	359.117		ESI		[128]	
Oleuropein aglycone derivative	C_16_H_26_O_10_	377.145		ESI	[7,100,103]	[130]	[134,136]
Oleuropein derivative	C_25_H_36_O_12_	527.213		ESI		[127]	
Oleuropein dimer	C_50_H_62_O_25_	1075.33		ESI		[130]	
Oleuropein glucoside isomers	C_31_H_42_O_18_	701.23	703.244 720.271 ^b^	APCI, ESI, FAB	[7] [93] ^4^ [94,99,100,101,102,103] [106] ^5^ [108]	[49,130,133]	[133,134]
Oleuropein trimer	C_75_H_90_O_39_	1613.499		ESI		[130]	
Oleuroside	C_25_H_32_O_13_	539.177	541.193	ESI	[94,98,99,101,102,103,108]	[49]	[134]
Oxidized isoverbascoside	C_29_H_34_O_15_	621.183		ESI			[135]
Oxidized verbascoside	C_29_H_34_O_15_	621.183		ESI			[135]
*p*-Coumaric acid	C_9_H_8_O_3_	163.04	165.055	APCI, ESI	[101,105] [106] ^5^	[49,127,131,133]	[133,136]
*p*-Coumaroyl aldarate	C_15_H_16_O_10_	355.067		ESI		[49]	
*p*-Coumaroyl hexoside	C_15_H_18_O_8_	325.093		ESI		[49]	
Phenylalanine	C_9_H_11_NO_2_	164.072		ESI		[127]	
*p-*Hydroxybenzoic acid (4-hydroxybenzoic acid)	C_7_H_6_O_3_	137.024	139.038	APCI, ESI	[101,103] [106] ^5^		[138]
Peonidin-3-*O*-rutinoside	C_28_H_33_O_15_^+^		609.181	ESI	[95]		
Pinoresinol	C_20_H_22_O_6_	357.134	359.149	APCI, ESI	[100,105] [106] ^5^ [109]	[50,127,131,132]	
Protocatechuic acid	C_7_H_6_O_4_	153.019	155.034	APCI, ESI	[105] [106] ^5^	[127]	[138]
Quercetin	C_15_H_10_O_7_	301.035	303.05	APCI, ESI	[98,99,103,105] [106] ^5^ [108,109]	[127]	[134]
Quercetin arabinose	C_20_H_18_O_11_	433.078		ESI		[128]	
Quercetin-4-*O*-glucoside	C_21_H_20_O_12_	463.088	465.101	APCI, ESI	[106] ^5^ [108]		
Quercetin-3-*O*-rhamnoside	C_21_H_20_O_11_	447.093	449.108	ESI, MALDI	[76] ^1^		[134]
Quinic acid	C_7_H_12_O_6_	191.056	193.071	APCI, ESI	[7,100,101] [106] ^5^	[49,132]	[136]
Quinone oxydized hydroxytirosol	C_8_H_8_O_3_	151.04	153.054	ESI		[132]	
Rutin	C_27_H_30_O_16_	609.146	611.161	APCI, ESI	[7] [9] ^3^ [23,94,99,100,101,102] [106] ^5^ [108]	[127,130,131,133]	[133,134,136]
Secologanic acid	C_18_H_26_O_10_	401.145		ESI		[127]	
Secologanin	C_17_H_24_O_10_	387.13		ESI		[127]	
Secologanoside (oleoside positional isomer)	C_16_H_23_O_11_	389.109		APCI, ESI	[7,23] [76] ^1^ [99,100,101,102,103] [106] ^5^ [108]	[127,132,133]	[133]
Shikimic acid	C_7_H_10_O_5_	173.05		APCI, ESI		[127]	
Syringaresinol	C_22_H_25_O_8_	417.156	419.169	APCI, ESI	[99,100] [106] ^5^		
Taxifolin	C_15_H_12_O_7_	303.051	305.066	APCI, ESI	[7,99,100,103,105] [106] ^5^	[127]	
Tyrosol	C_8_H_10_O_2_	137.061	121.064 ^a^	APCI, ESI	[23] [106] ^5^ [109]	[49,50,127,131,133]	[133,136,138,139]
Tyrosol glucoside	C_14_H_20_O_7_	299.114	301.13	APCI, ESI	[102] [106] ^5^	[127,133]	[133,134]
Vanillic acid	C_8_H_8_O_4_	167.035		ESI	[105]	[127,131,132,133]	[133,138]
Vanillic acid hexoside	C_14_H_18_O_9_	329.088		ESI		[49]	
Vanillin	C_8_H_8_O_3_	151.04		ESI	[7,100,101,103,109]	[131]	
Verbascoside	C_29_H_36_O_15_	623.198	625.213	APCI, ESI	[7,23,94,98,101,102,105] [106] ^5^ [108]	[49,96,127,128,130,131,133]	[133,134,135,136]

^1^ MALDI-MS, ^2^ FAB-MS; ^3^ APCI-MS; ^4^ ESI-MS and FAB; ^5^ ESI-MS and APCI; ^a^ [M + H-H_2_O]^+^, ^b^ [M + NH_4_]^+^, ^c^ [M + Na]^+^, ^d^ [M + H-H_2_O].

**Table 3:** Range of concentrations of bioactive compounds reported in leaves (mg/g), olive pomace (mg/kg) and in olive oil mill wastewaters (mg/kg) (OOMW).

Name	Leaves	Refs.	OP	Refs.	OOMW	Refs.
3,4- dihydroxyphenylglycol			7.50–20.16	[150]		
Oleoside glucoside (6′-β- glucopyranosyl-oleoside)			5.0–5.0	[130] ^b^		
6′-β-rhamnopyranosyl-oleoside			6.4–6.6	[130] ^b^		
Acteoside	7.8–9.2	[103]				
Acetoxypinoresinol			9.32–12.2 14.4–15.2	[149] [132]		
Apigenin	0.06–0.12	[105]	0.195–0.501 11.4–13.6 19.5–25.5 29.7–29.9	[149] [151] [96] [132]	2.5–6.5	[151]
Apigenin-7-*O*-glucoside	0.197–0.217 0.230–0.386 0.33–0.36	[96] [102] [105]	5.3–7.1	[96]		
Apigenin-7-*O*-rutinoside			0.7–0.9	[151]		
Caffeic acid	0.012–0.018 0.60–0.66 1.0–1.2	[105] [103] [34]	6.7–13.5 67–69	[147] [49] ^a^	19.2–57.3 0.014–0.017	[151] [5] ^d^
Chlorogenic acid (Caffeoyl-quinic acid)	0.00187–0.00347	[105]				
Chrysoeriol-7-*O*-glucoside	0.581–0.845	[102]				
Demethyloleuropein	1.338–6.382	[102]	11.2–22.4	[151]		
Elenolic acid (EA)			2.1–2.6	[151]	4.9–11.7	[151]
Elenolic acid (EA) derivative (decarboxylated form of hydroxyelenolic acid)			59–61 153–601	[132] [149]		
Elenolic acid (EA) derivative			788–896	[150]		
Elenolic acid decarboxymethylated (EDA)			515–601	[150]		
Elenolic acid glucoside (Oleoside methyl ester)	0.267–1.367	[102]	32.9–33.9	[132]		
Eriodictyol	0.0062–0.0074	[105]				
Ferulic acid	0.035–0.055 15–37	[105] [34]	12.6	[147]		
Gallic acid	0.0020–0.0032 0.3–1.8	[105] [34]	0.0–1.6 11.4–12.6	[151] [147]	3.86–6.71 22.2–61.0	[138] [151]
Hydro-oleuropein			99–101	[132]		
Hydroxytyrosol (3,4-DHPEA)	6–24 18.3–20.1	[34] [97]	8.4–10.4 60–163 159–181 799–1059 809–853	[147] [58] [149] [150] [96]	157.2–245.1 483.0–1733.2 544–1560 1230–1290 0.25–18.2 0.0020–0.1224	[151] [138] [80] [77] [139] ^c^ [5] ^d^
Hydroxytyrosol acetate	16.6–18.8	[97]	44–167 562–866	[58] [150]		
Hydroxytyrosol diglucoside			87.0–89.6	[49] ^a^		
Hydroxytyrosol glucoside	0.340–0.793	[102]	164.0–166.4 6.2–6.8	[49] ^a^ [130] ^b^	1300 -1700	[77]
Ligstroside	3.251–3.845	[102]	2.5–2.9 15.7–17.3	[151] [132]	0.0087–0.0092	[5] ^d^
Luteolin	0.216–0.224 0.367–0.497 1.16–1.30 10.4–53.5	[105] [102] [103] [97]	11.3–147.3 14.3–32.7 19.3–25.5 217–225	[149] [151] [96] [132]	2.5–36.2 270–510 0.0145–0.021	[151] [77] [5] ^d^
Luteolin diglucoside	0.201–0.364	[102]	3.2–3.2	[151]		
Luteolin-4′-*O*-glucoside	70.1–71.1	[97]	0.46–0.48	[130] ^b^		
Luteolin-7-*O*-glucoside	0.145–0.165 2.16–2.34 5.6–6.6 68–90	[96] [105] [45] [97]	12.0–16.6 19–23 2.1–2.1	[96] [132] [130] ^b^	0–0.0214	[5] ^d^
Luteolin-7-*O*-rutinoside			1.0–1.2 2.2–2.4	[151] [132]		
Luteolin-hexoside	1.072–1.744	[102]			3.2–24.2	[151]
Luteolin-*O*-rutinoside	0.199–0.491	[102]	0.31–0.34	[130] ^b^		
Methoxyloleuropein	0.870–2.188	[102]				
Oleacin (3,4-DHPEA-EDA)			2.5–3.0	[151]	11300–45951	[80]
Oleoside	0.366–0.399	[102]	3.4–3.7	[130] ^b^		
Oleuropein	0.13–21.34 17.1–18.1 18.2–19.9 52–58 60–90 83–87 92.5–345.3	[95] [102] [96] [45] [122] [103] [97]	1.3–11.0 7.62–14.67 33–41 81.7–83.0 93–95	[151] [150] [96] [147] [132]	5.7–27.0 420–560	[151] [77]
Oleuropein aglycone (3,4-DHPEA-EA)	0.134–0.288	[102]	23.3–24.0 23.5–48.0	[147] [149]		
Oleuropein aglycone derivative			45.7–152.8	[149]		
Oleuropein derivative	0.648–1.948	[34]	27.6–39.2	[150]	5400–7600	[77]
Oleuroside	2.100–2.337	[102]	0.4–1.4	[151]	200–400	[77]
*p*-Coumaric acid	0.072–0.090 0.9–5.6	[105] [34]	0.8–0.9 17.2–18.2	[151] [49] ^a^	15.9–21.8	[151]
Ligstroside aglycone (*p*-HPEA-EA)			0.3–10.2 27.1–31.1	[151] [147]		
Oleocanthal (*p*-HPEA-EDA)			1.9–2.3 62.4–128.3	[151] [148]		
*p-*Hydroxybenzoic acid (4-hydroxybenzoic acid)	0.0143–0.0163 0.9–4.2	[105] [34]	6.86–13.80	[150]	1.75–6.15	[138]
Pinoresinol	0.0033–0.0047	[105]	2.6–3.2 17–137	[132] [58]		
Protocatechuic acid	0.013–0.021 32–37	[105] [34]	1.3–3.8 38.4–44.4	[151] [150]	2.77–5.29 25.3–136.7	[138] [151]
Quercetin	0.037–0.043	[105]				
Quinic acid			203–243 529–1485	[132] [149]		
Rutin	0.289–0.651	[102]	0.63–0.69 1.1–1.5	[130] ^b^ [151]	440–640	[77]
Secologanoside (oleoside positional isomer)	1.823–3.677	[102]	34–46	[132]		
Taxifolin	0.0070–0.0094	[105]				
Tyrosol	3–14	[34]	20.7–21.6 34–71 69.6–196.7 96–124	[147] [58] [150] [149]	218.4–581.0 1180–1560 0.19–4.32 0.0145–0.0208	[138] [77] [139] ^c^ [5] ^d^
Tyrosol glucoside	0.863–1.278	[102]	6.6–12.1	[151]		
Vanillic acid	0.010–0.020	[105]	1.3–1.7 8.8–10.4 20.6–25.2	[151] [147] [150]	1.68–62.7 0.0174–0.0198	[138] [5] ^d^
Vanillin			140–150	[149]		
Verbascoside	1.127–4.069 1.382–1.474 1.71–2.09 2.730–3.090	[102] [96] [45] [105]	0.4–1.6 17.6–23.0	[151] [96]	14496–24100 1620–1760 0.0075–0.0155	[80] [77] [5] ^d^

Content expressed as mg/L ^a^, mg/g ^b^, mg/mL ^c^, and weight% ^d^.

## Data Availability

Not applicable.
